# Corrosion Resistance of Aluminum Alloy AA2024 with Hard Anodizing in Sulfuric Acid-Free Solution

**DOI:** 10.3390/ma15186401

**Published:** 2022-09-15

**Authors:** José Cabral Miramontes, Citlalli Gaona Tiburcio, Estefanía García Mata, Miguel Ángel Esneider Alcála, Erick Maldonado-Bandala, Maria Lara-Banda, Demetrio Nieves-Mendoza, Javier Olguín-Coca, Patricia Zambrano-Robledo, Luis Daimir López-León, Facundo Almeraya Calderón

**Affiliations:** 1 Universidad Autónoma de Nuevo León, FIME-Centro de Investigación e Innovación en Ingeniería Aeronáutica (CIIIA), Av. Universidad s/n, Ciudad Universitaria, San Nicolás de los Garza 66455, Mexico; 2 Centro de Investigación en Materiales Avanzados Subsede Monterrey (CIMAV), Alianza Norte 202, PIIT, Autopista Monterrey-Aeropuerto, Km 10, Apodaca, Nuevo León 66628, Mexico; 3 Facultad de Ingeniería Civil, Universidad Veracruzana, Xalapa 91000, Mexico; 4 Área Académica de Ingeniería y Arquitectura, Universidad Autónoma del Estado de Hidalgo, 42082 Carretera Pachuca-Tulancingo, Km 4.5, Hidalgo 42082, Mexico

**Keywords:** aluminum alloy, hard coatings, corrosion, environmentally friendly

## Abstract

In the aeronautical industry, Al-Cu alloys are used as a structural material in the manufacturing of commercial aircraft due to their high mechanical properties and low density. One of the main issues with these Al-Cu alloy systems is their low corrosion resistance in aggressive substances; as a result, Al-Cu alloys are electrochemically treated by anodizing processes to increase their corrosion resistance. Hard anodizing realized on AA2024 was performed in citric and sulfuric acid solutions for 60 min with constant stirring using current densities 3 and 4.5 A/dm^2^. After anodizing, a 60 min sealing procedure in water at 95 °C was performed. Scanning electron microscopy (SEM) and Vickers microhardness (HV) measurements were used to characterize the microstructure and mechanical properties of the hard anodizing material. Electrochemical corrosion was carried out using cyclic potentiodynamic polarization curves (CPP) and electrochemical impedance spectroscopy (EIS) in a 3.5 wt. % NaCl solution. The results indicate that the corrosion resistance of Al-Cu alloys in citric acid solutions with a current density 4.5 A/dm^2^ was the best, with corrosion current densities of 2 × 10^−8^ and 2 × 10^−9^ A/cm^2^. Citric acid-anodized samples had a higher corrosion resistance than un-anodized materials, making citric acid a viable alternative for fabricating hard-anodized Al-Cu alloys.

## 1. Introduction

In the aeronautical industry, aluminum alloys are used for their low cost and combination of properties [1]. Since their invention in 1825, aluminum alloys have been known to provide significant benefits to the aeronautical industry. They are, thus, one of the most used materials for aircraft structures where fatigue and corrosion resistance are required [2,3]. The 2XXX (Al-Cu) series is one of the most used for fabricating structures that are sensitive to fatigue, damage tolerant, and require a high specific resistance. AA2024 alloy is used in the fuselage (bulkheads and longerons), internal structures (trusses), and non-structural components [4,5,6,7]. The 2XXX (Al-Cu) series aluminum has mainly Cu in its chemical composition and is used for its superior mechanical properties. However, the presence of the high Cu content results in corrosion problems due to poor anodic coating quality [3,8]. In addition, the aluminum alloy AA2024 presents Cu, Fe, Si, Mn, and Mg, among other elements [9]. Additionally, the humidity in the air can form reactive atomic hydrogen in aluminum alloys. As a result, a significant element impacting the mechanical characteristics of the metal can be the relative humidity (RH) in the atmosphere [10]. The main advantage of AA2024 for aircraft structural components is that it provides good fatigue resistance and lower corrosion resistance, as mentioned previously [8]. Nevertheless, AA2024 can also be found in general aircraft parts including shear webs and ribs, gears, bolts, couplings, hydraulic valves, pistons, avionics, etc. [11]. AA2024 is a trusted material because of its tensile strength, achieving up to 483 MPa, and elasticity modulus of 73.1 GPa. All these properties make the Al alloys a good option for any conditions [12].

Furthermore, the anodizing process is the most popular and cheapest option to improve corrosion resistance and achieve better properties for aluminum alloys [1,13]. Anodizing is an electrochemical process by which several conditions influence the mechanical properties, chemical composition, and morphology by creating an anodic oxide film on the aluminum alloy’s surface [14,15]. The resulting properties of the oxide film are thickness, hardness, and elasticity, which can vary depending on the electrolyte used in the anodizing cell [16]. However, several factors affect the anodizing process, such as the type of aluminum alloy, the processing time, the concentration of each solution, temperature, potential applied to the cell, current density, sealing process, etc. [17,18] Some advantages of the anodizing process are increasing corrosion and abrasion resistance, improving paint adhesion, and creating a dielectric layer [14]. One of the most important anodizing methods for AA2024 in aeronautical applications is hard anodizing (hard coats or type III anodizing). Hard anodizing produces a hard anodic film on the aluminum alloy by considering the operating temperatures, use of addition agents, and the voltage and current density, which differ from the common sulfuric acid method or type II. The hard anodizing creates a heavier coating, expected to be up to >3 GPa at 25 µm film thickness [14,18,19]. This process is commonly given by a pretreatment process, where the alloys can be degreased with acetone and rinsed in deionized water. Following that, etching proceeds in the acid bath. Finally, the anodizing treatment is performed in the desirable solutions (inorganic acids) and sealed at high temperatures (up to 90 °C) [20,21]. Nevertheless, it is crucial to keep in mind that the hard coatings are exposed to form linear pores and cells in the morphology of the anodic film while enhancing corrosion resistance and proper paint adhesion [22].

Over the decades, optimization of the anodizing process has been needed to minimize every type of corrosion and reach the expected product lifetime [15]. However, it has been a challenging demand since the expectations must also meet the environmental standards while preserving energy efficiency [23]. Unfortunately, for most type III hard coatings, it is common to use sulfuric and chromic acid solutions, which are hazardous to human health [24,25]. In contrast, several studies show that the higher the acid concentration, the greater the thickness in the anodic film [26], leading to the minimizing of sulfuric and chromic acid. In addition, organic acids have proven to be the best options for replacing the inorganic ones [27], citric acid being one of the most predominant and having an overall great performance in aluminum alloy. At the same time, higher concentrations are not needed to provide protection and better properties in the anodic film [24,28]. This work aims to develop a surface treatment for the manufacture of type III hard anodizing free chromic and sulfuric acid solutions in Al-Cu alloys and to evaluate its corrosion resistance and microhardness properties.

## 2. Materials and Methods

### 2.1. Materials

A commercial AA2024 alloy in the shape of a 50-mm-diameter rod was used, and 5-mm-thick discs were cut to anodize and evaluate using electrochemical techniques.

AA2024 samples were polished per ASTM E3 and E407 standards [29,30] prior to anodization with SiC grit papers: 180, 220, 320, 400, and 600 grades, followed by 10 min of ultrasonic cleaning in deionized water.

### 2.2. Anodizing Process

Anodizing procedure included 5 s of pickling in a 50 wt. % HCl solution, followed by rinses in deionized water. AA2024 was anodized using mix of bath solutions, which included the following: 1 M sulfuric acid as a control, a solution composed of a 1 M citric acid concentration with additions of 5 mL/L and 10 mL/L of the sulfuric acid solution, and finally a bath composed of a concentration of 1 M citric acid (C_6_H_8_O_7_·H_2_O) to give a total of four solutions used to produce anodized AA 2024. It used a lead bar as a cathode because they have a more noble behavior than aluminum alloy AA2024, and a Model XLN30052-GL High Power Programmable DC Power Supply (CA, USA) was used as the current generator. The parameters used in the anodizing process were the anodizing electrolyte baths and the current densities of 3 and 4.5 A/dm^2^ [31]. The anodization was carried out in a cold-water bath at a temperature of 0 °C ± 2 °C for 60 min for each of the solutions individually with constant stirring of the solution. The anodizing current density and time strongly depend on the electrolyte species used during anodizing, to grow such a thick oxide, current densities can be markedly higher than those used to grow type II oxides, depending upon how the components are manufactured and the complexity of the alloy. After anodizing, a 5 s rinse in deionized water was followed by 60 min sealing in deionized water at 95 °C. Hot-water sealing reduces or eliminates porosity in anodizing and improves corrosion resistance [32]. Parameters of the anodizing treatment are shown in Table 1.

### 2.3. Microstructural Characterization

Microstructural characterization was performed by scanning electron microscope (SEM, Jeol JSM 6510LV, Tokyo, Japan) to analyze the surface morphology and thickness of oxide layer (cross-section) of the anodized samples at a magnification of 2000×, a voltage of 20 kV, and a working distance (WD) of 8.5 mm. Backscattered electrons (BSE) were used to observe the morphology by SEM, and energy dispersive X-ray spectroscopy (EDS) was used to determine the chemical composition of cross-sections.

### 2.4. Vickers Microhardness Measurements

Vickers microhardness measurements were made in cross-section of anodized samples in a microhardness tester (Wilson Tester 402 MVD, Lake Bluff, IL, USA) using a load 0.05 gf and a dwell time 15 s, with 15 readings per sample.

### 2.5. Electrochemical Techniques

Electrochemical corrosion measurements were performed using a three-electrode cell, and a Potentiostat/Galvanostat/ZRA (Solartron 1287A, Bognor Regis, UK). All tests were by immersion in a 3.5 wt. % sodium chloride solution at room temperature and were performed in triplicate. Electrochemical techniques used were cyclic potentiodynamic polarization (CPP) and electrochemical impedance spectroscopy (EIS) [33,34]. CPP was measured with a potential scan from −0.3 to 1.0 V vs. SCE from corrosion potential (Ecorr) using a complete polarization cycle at a sweep rate of 0.06 V/min following the ASTM G61-11 standard [35,36,37,38,39,40]. EIS has measured a frequency range of 0.01 to 100,000 Hz, obtaining 35 points per decade and using a 10 mV RMS amplitude by the ASTM G106-15 standard [41,42,43,44,45,46,47,48].

## 3. Results

### 3.1. Microstructural Characterization by SEM

#### 3.1.1. Surface Morphology

**Figure 1:**
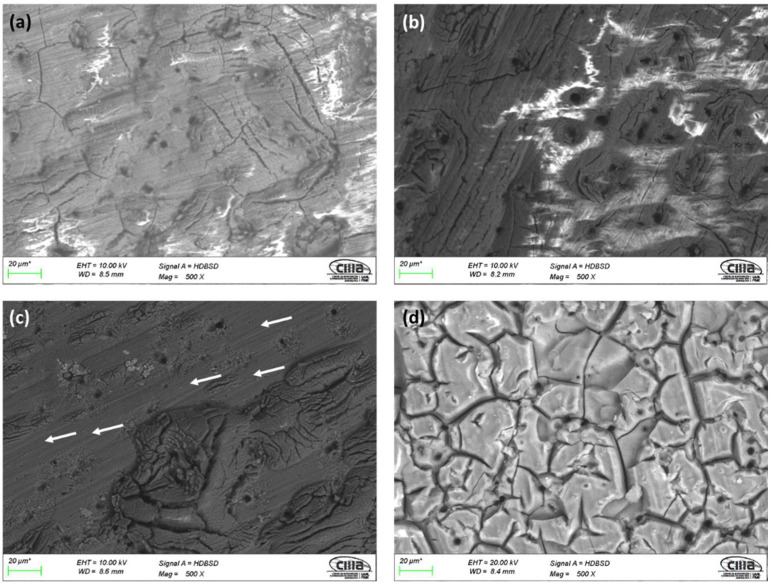
SEM-BSE surface morphology of anodized samples of AA2024 at 3 A/dm^2^: (**a**) 3A C1M S5, (**b**) 3A C1M S10, (**c**) 3A C1M, and (**d**) 3A S1M.

**Figure 2:**
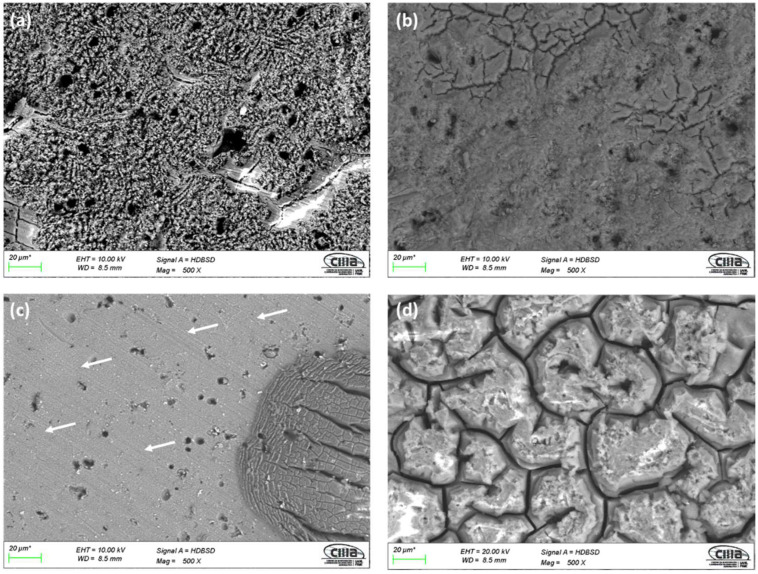
SEM-BSE surface morphology of anodized samples of AA2024 at 4.5 A/dm^2^: (**a**) 4.5A C1M S5, (**b**) 4.5A C1M S10, (**c**) 4.5A C1M, and (**d**) 4.5A S1M.

Surface morphology obtained by SEM using backscattered electrons (BSE) for anodized samples can be seen in Figure 1 and Figure 2. All the samples prepared with an i = 3 A/dm^2^ and the anodizing solutions have porosity and surface cracks. The sample anodized in 1 M citric acid solution (3A C1M) shows areas without anodizing, indicated by arrows in Figure 1c, as well as surface porosity and cracking. In all cases, anodized samples with an i = 4.5 A/dm^2^ in the various solutions exhibited the same behavior, cracking and surface porosity. Similarly, the anodized sample in 1 M citric acid solution (4.5A C1M) has areas that are not anodized, as indicated by the arrows in Figure 2c. In all cases, the porosity may be related to the intermetallic copper compounds, which do not allow a homogeneous growth of the anodizing layer; on the other hand, the cracking may be due to surface stresses caused by the growth of the anodizing film or to thermal shock that occurs when going from a cold-water bath in the anodizing process to a bath at 95 °C for the sealing process.

#### 3.1.2. Morphology of Cross-Sections

**Figure 3:**
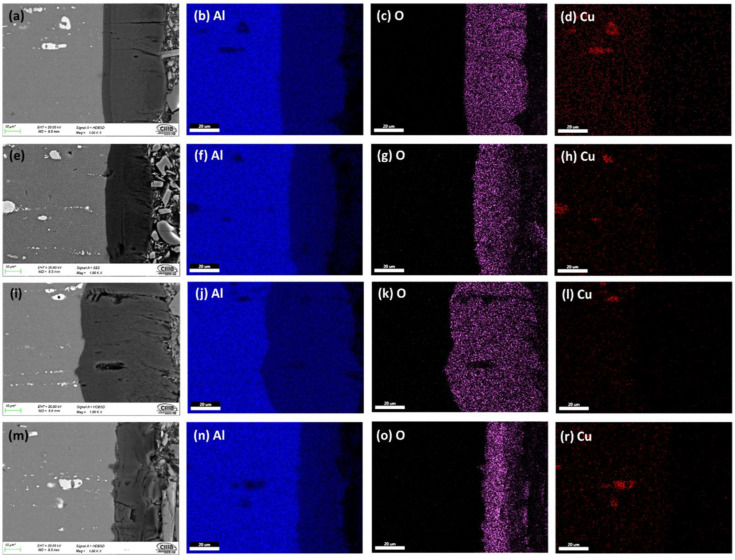
SEM-BSE cross-section of anodized samples of AA2024 at 3 A/dm^2^: (**a**) 3A C1M S5, (**e**) 3A C1M S10, (**i**) 3A C1M, and (**m**) 3A S1M. Element content; Aluminum (**b**,**f**,**j**,**n**), Oxygen (**c**,**g**,**k**,**o**), and Copper (**d**,**h**,**l**,**r**).

**Figure 4:**
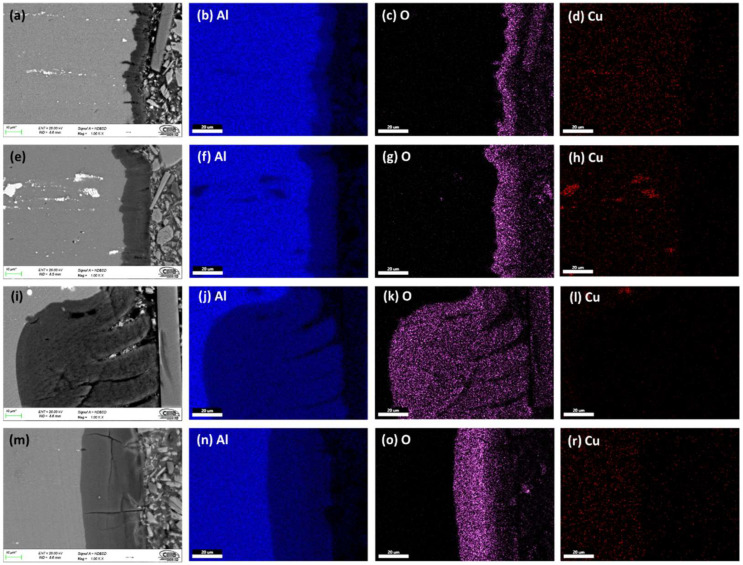
SEM-BSE cross-section of anodized samples of AA2024 at 3 A/dm^2^: (**a**) 4.5A C1M S5, (**e**) 4.5A C1M S10, (**i**) 4.5A C1M, and (**m**) 4.5A S1M. Element content; Aluminum (**b**,**f**,**j**,**n**), Oxygen (**c**,**g**,**k**,**o**), and Copper (**d**,**h**,**l**,**r**).

Figure 3 and Figure 4 show the cross-section micrographs obtained by SEM and mapping of the chemical composition obtained by EDS of the anodized AA2024 alloy under different conditions. Figure 3a,e,i,m show that the anodized coating layer has porosity and cracking, which in some cases extends from the coatings surface to the base material. In sample 3 A S1M, there is also cracking. However, this does not reach the base material’s surface, as seen in Figure 3m.

The mapping of the chemical composition of the anodizing coating by EDS showed the aluminum content of the base material (substrate), in Figure 3b,f,j,n. A high concentration of oxygen in Figure 3c,g,k,o corresponds to the Al_2_O_3_ layer formed during the anodizing process. The white dots (see Figure 3a,e,i,m) correspond to Al_2_Cu precipitates typical of this type of alloy from the 2XXX series of aluminum alloys [49].

On the surface of the anodized samples, 4.5A C1M S5 and 4.5A C1M S10 with i = 4.5 A/dm^2^, porosity and cracking were not observed (Figure 4a,e). Porosity and cracking that could reach the substrate from the surface was observed in the samples 4.5A C1M and 4.5A S1M (Figure 4i,m). In Figure 4b,f,j,n, it can be observed by mapping of the chemical elements that have the presence of a higher concentration of aluminum in the base material, that the said concentration of aluminum decreases in the anodized coating. Figure 4c,g,k,o clearly show the formation of the Al_2_O_3_ layer derived from the type III hard anodizing process due to the high concentration of oxygen present. The Al_2_Cu precipitates can be seen in Figure 4d,h,l,r.

**Figure 5:**
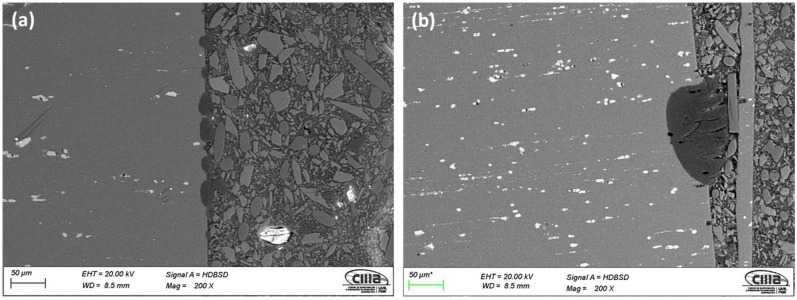
SEM-BES cross-section micrographs of anodized samples of AA2024: (**a**) 3A C1M and (**b**) 4.5A C1M.

It can be seen in Figure 3e,m and Figure 4e,m that these coatings are homogeneous and show less cracking than the other samples manufactured with the two anodizing current densities 3 and 4.5 A/dm^2^. Samples 3A C1M and 4.5A C1M have areas without anodizing, as shown in Figure 5, at a lower magnification (200×). This is because some studies have found that alumina incorporated with citric acid takes a long time to deposit on the entire surface of the alumina. Once the pores nucleate and grow, the equilibrium self-organization process begins where the thickness of the barrier layer is reduced [50,51]. Additionally, it has been reported that to grow the anodic layers in electrolytes composed of organic acids such as citric and malonic, voltages greater than 350 V, temperatures below 0 °C, and anodizing times of up to 5 h are needed [52,53].

#### 3.1.3. The Thickness of Anodized Samples of AA2024

**Figure 6:**
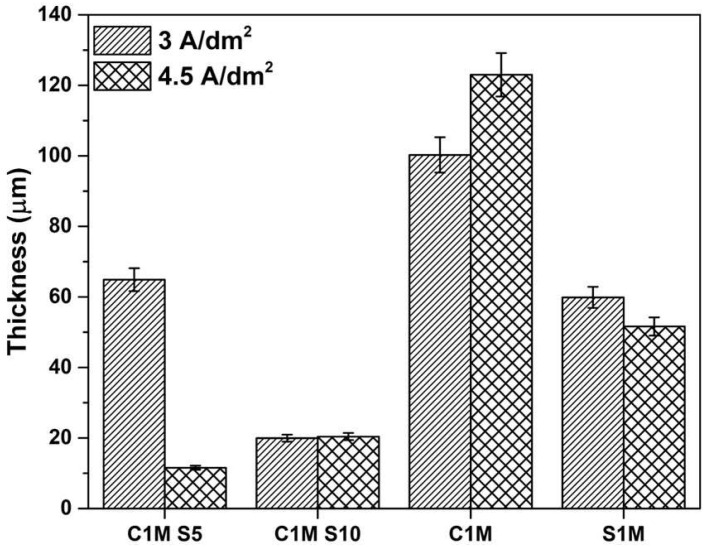
Thicknesses obtained vs. anodized samples of AA2024 at 3 and 4.5 A/dm^2^.

Figure 6 shows the thickness obtained by SEM in cross-sectional anodized samples of AA2024 at i = 3 and 4.5 A/dm^2^ in different citric and sulfuric acid bath solutions. Anodized samples 3A C1M S5, 3A C1M S10, and 4.5A C1M S10 presented lower coating thicknesses, with 11.57, 19.95, and 20.41 µm. The rest of the coatings presented thicknesses greater than 50 µm, reaching 120 µm in some cases.

Samples 3A C1M and 4.5A C1M presented the greatest thicknesses. However these samples presented areas without anodizing (Figure 1c, Figure 2c, and Figure 5). Thin coatings form when an additive prevents the oxide coating from dissolving.

The thickness varies due to the applied current density as it affects the growth rate of oxidation films. Most of the thicknesses obtained in the various anodizing conditions allowed thicknesses greater than 12.7 m (minimum thickness required by MIL-A-8625 standard) [54].

### 3.2. Vickers Microhardness Measurements

**Figure 7:**
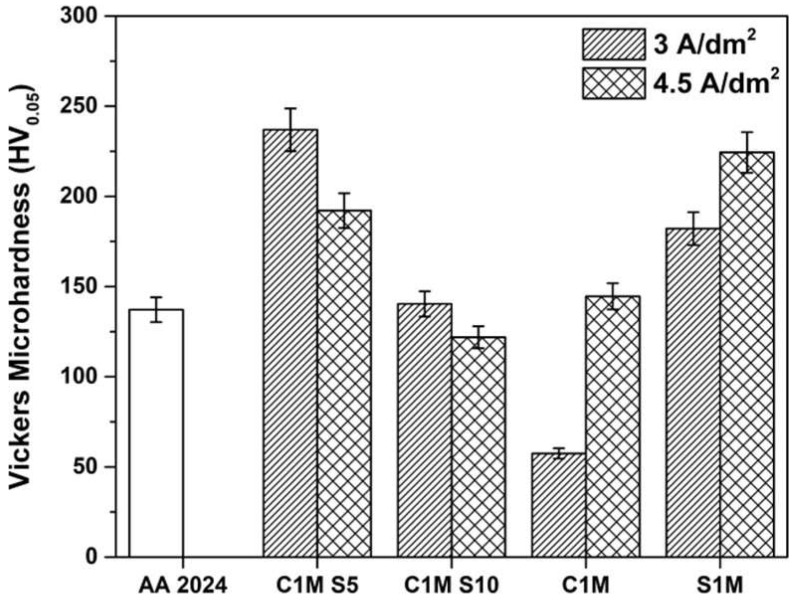
Vickers microhardness measurements of non-anodized and hard anodizing AA2024 samples at 3 and 4.5 A/dm^2^.

Vickers microhardness measurement results are presented in Figure 7. The highest microhardness values were found in samples 3A C1M S5, 4.5A C1M S5, and 4.5A S1M, with 236, 192, and 224 HV values, respectively. Samples 4.5A C1M S5, 3A C1M S10, 4.5A C1M, and 3A S1M presented higher hardness values than those shown by the base material AA2024 (137 HV). The samples with the lowest hardness were 3A C1M and 4.5A C1M S10, with values of 57 and 121 HV, respectively.

Hardness values from 150 to 300 HV in this work can be associated with the formation of Bayerite (Al_2_O_3_·3H_2_O) [55]. The low hardness values presented by samples 3A C1M and 4.5A C1M S10 may be associated with the high concentrations of baths, high temperatures, and prolonged processing times [54]. In this work, Vickers microhardness values higher than 300 HV were not obtained, which are common values for type III hard anodizing [1].

### 3.3. Electrochemical Techniques

#### 3.3.1. Cyclic Potentiodynamic Polarization

The CPP curves analyzed the corrosion process, which indicates the information of the anodic and cathodic branches and the hysteresis curve of the anodized samples.

**Figure 8:**
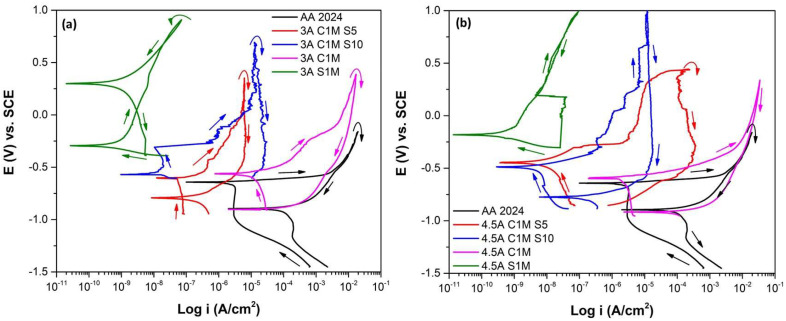
Cyclic potentiodynamic polarization curves of anodized samples of AA2024 immersed in NaCl solution. (**a**) i = 3 A/dm^2^ and (**b**) i = 4.5 A/dm^2^.

Figure 8 shows the corrosion behavior of samples in different solutions with an i = 3 and 4.5 A/dm^2^ immersed in 3.5 wt. % NaCl solution. Figure 8a shows that AA2024 without anodizing has the lowest corrosion potential (E_corr_). All anodized samples showed more electropositive E_corr_. The 3A C1M sample presented an E_corr_ = −0.600 mV, slightly higher than the potential of the AA2024 sample because it presented areas without anodizing, as observed in Figure 1c. Sample 3A S1M reached an E_corr_ = −0.293 mV, the highest of the anodized samples with current density 3 A/dm^2^. Nobler values of E_corr_ in anodized samples indicate less corrosion susceptibility [56,57]. Pitting potential (E_pit_) is the potential value at which the current increases and the pitting attack occurs.

Only sample 3A C1M S10 presented an E_pit_ = −0.308 mV; in the rest of the samples, E_pit_ did not appear. This behavior can be due to two reasons, the anodized surface is imperfect, or because of the breakdown of the oxide film and the appearance of pitting corrosion in the presence of aggressive ions such as Cl^−^.

When there is an oxide film on the material surface prior to polarization, the pitting potential coincides with the corrosion potential for some materials. Because there is an intersection between the anodic branch (transpassive region) and the cathodic branch, the corrosion potential will be the same as the pitting potential [58]. The anodic to cathodic transition potential (E_A–C_) defines the necessary potential where the anodic current density varies with the cathodic current density. Only sample 3A S1M presented a nobler E_A–C_ than E_corr_, which indicates that the sample is not susceptible to pitting corrosion. For samples 3A C1M S5, 3A C1M, and AA2024, the E_A–C_ is more negative than E_corr_. Therefore, the protection of the oxide layer will persist [59,60]. In the sample, 3A C1M S10 E_A–C_ was not present. The hysteresis loops were positive in most anodized samples with an i = 3 A/dm^2^, indicating localized corrosion. With this current density, only sample 3A S1M presented negative hysteresis; therefore, this sample has resistance to localized corrosion (Figure 8a). The difference in the i_corr_ of sample 3A C1M S5 (1.57 × 10^−7^ A/cm^2^) and 3A S1M (1.36 × 10^−10^ A/cm^2^) is mainly due to the morphology and characteristics of the generated anode layer (see Table 2). On the one hand, sample 3A C1M S5 presents porosity and cracking that reaches from the surface of the coating to the substrate, which causes the electrolyte to enter the base material, while, in sample 3A S1M, the cracking occurs laterally, which prevents the penetration of the electrolyte into the substrate, as can be seen in Figure 3a,m.

Figure 8b shows CPP for samples with an i = 4.5 A/dm^2^ immersed in a 3.5 wt. % NaCl solution. All the anodized samples with an i = 4.5 A/dm^2^ in the different baths have a nobler corrosion potential than the non-anodized material. As with the 3A C1M sample, the 4.5A C1M sample presented an E_corr_ = −0.593 mV, which is slightly higher than the potential of the AA2024 sample (E_corr_ = −0.656 mV) since it also has areas without anodizing, which can be seen in Figure 2c.

The corrosion potential of the 4.5A S1M was the highest of all the samples (E_corr_ = −0.180 mV), indicating less corrosion susceptibility. The 4.5A C1M S5 and 4.5A C1M S10 samples show an E_pit_ = 0.334 and 0.034 mV, respectively, vs. SCE.

E_pit_ present in some samples (3A C1M S5, 3A C1M S10, 3A C1M, 4.5A C1M S5, and 4.5A C1M S10) due to two reasons; the first is that the surface of the generated oxide is not perfect since there are some defects in coating, and the second could be due to the rupture of the oxide film and pitting corrosion appearance with the ions’ aggressive presence.

Sample 4.5A S1M is susceptible to pitting corrosion because it has a nobler E_A–C_ than E_corr_. The protection provided by the oxide layer persists in samples 4.5A C1M S10 and 4.5A C1M since the E_A-C_ is more negative than E_corr_ [61,62]. In samples 4.5A C1M S5 and 4.5A S1M, E_A–C_ did not appear. Localized corrosion occurs since the samples 4.5A C1M S5, 4.5A C1M S10, and 4.5A C1M show positive hysteresis loops; meanwhile, in the sample 4.5A S1M, there is generalized corrosion because its hysteresis loop is negative (Figure 8b).

The corrosion parameters obtained from the CPP (see Table 2), are E_corr_, E_pit_, E_A–C_, passivation current density (i_pass_), corrosion current density (i_corr_), and hysteresis type. The passivation current density was lower in samples 3A S1M and 4.5A S1M with values of 6.77 × 10^−9^ and 1.87 × 10^−8^ A/cm^2^, respectively; however, these samples were made in a conventional H_2_SO_4_ bath. However, samples 3A C1M S10, 4.5A C1M S5, and 4.5A C1M S10, also showed low i_pass_ =1.38 × 10^−8^, 7.52 × 10^−6^, and 2.44 × 10^−6^ A/cm^2^, respectively; however, only sample 3A C1M S10 reached values as low as samples anodized in conventional H_2_SO_4_ solution. The values obtained from the i_pass_ indicate that hard anodizing with citric acid solutions provides protection against corrosion that is not as effective as conventional anodizing in H_2_SO_4_ but is better than the material without anodizing treatment. This behavior has also been observed in anodized AA7075 alloys with other baths, and it is due to the barrier effect provided by the anodizing layer [63,64,65,66]. The i_corr_ is directly related to the corrosion resistance of different materials. In this work, the lowest i_corr_ was obtained in samples 3A S1M and 4.5A S1M, which were anodized in 1 M sulfuric acid solution.

The lower i_corr_ appeared in samples with 10 mL/L of the sulfuric acid solution in the bath. In samples with 5 mL/L of sulfuric acid, galvanic pairs appeared. Finally, in samples 3A C1M and 4.5A C1M, in which the anodizing solution was completely composed of citric acid (192 g in 1 L of water), the higher i_corr_ may be related to the formation of galvanic pairs between the anodized areas (cathodic sites) and non-anodized areas (anodic sites) (see Table 2).

#### 3.3.2. Electrochemical Impedance Spectroscopy Measurements

**Figure 9:**
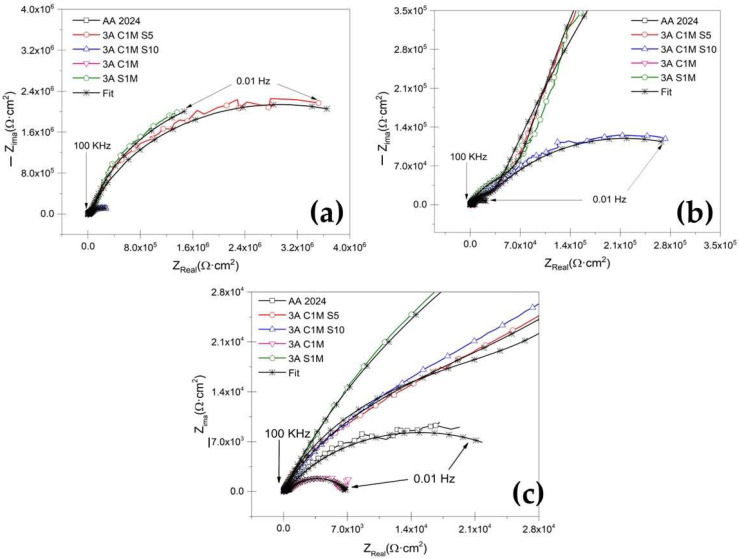
Nyquist plot for un-anodized AA2024 and citric–sulfuric acid-anodized samples immersed in NaCl solution. (**a**) i= 3 A/dm^2^, (**b**) first zoom of I = 3 A/dm^2^, and (**c**) second zoom of i = 3 A/dm^2^.

Figure 9a shows the Nyquist plot for anodized samples at i = 3 A/dm^2^ current density and different solutions immersed in 3.5 wt. % NaCl solution. The sample without anodizing treatment, AA2024, shows a characteristic behavior for aluminum alloys with an oxide layer naturally formed on the surface when in contact with oxygen (Figure 9c) [63,64,65,66]. For anodizing with the various mixtures of citric–sulfuric acid and a current density of 3 A/dm^2^ (Figure 9b,c), there is a high-frequency semicircle and a capacitive behavior at lower frequencies, which correspond to the characteristics of the porous layer and the barrier layer, respectively. In the zoom of Figure 9b,c, it can be seen that samples 3A C1M S10 and 4.5A C1M S5 present this same behavior.

**Figure 10:**
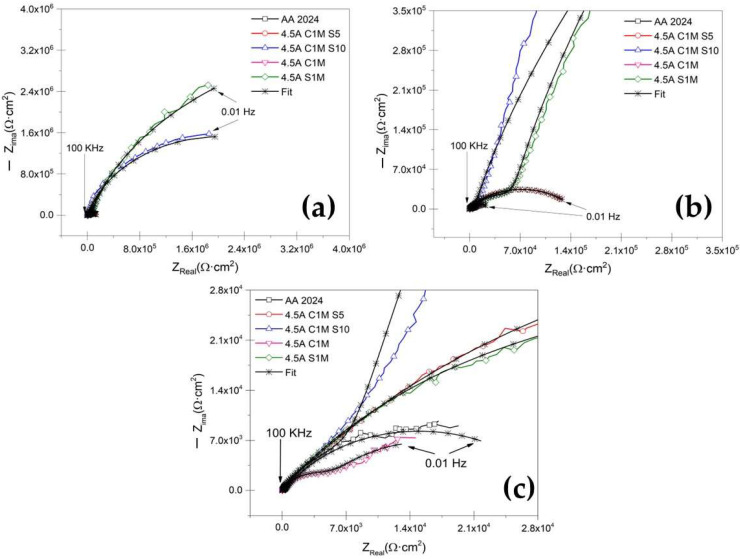
Nyquist plot for un-anodized AA2024 and citric–sulfuric acid-anodized samples immersed in NaCl solution. (**a**) i = 4.5 A/dm^2^, (**b**) first zoom of i = 4.5 A/dm^2^, and (**c**) second zoom of i= 4.5 A/dm^2^.

Similarly, Figure 10a shows the Nyquist plot obtained for anodized samples in a mixture of citric–sulfuric acid and a current density of 4.5 A/dm^2^, exposed to the 3.5 wt. % NaCl electrolyte. In this case, the anodized samples with a current density of 4.5 A/dm^2^ in all the solutions composed of citric–sulfuric acid present the characteristics of the porous layer in the high-frequency range, while in the low-frequency range they often exhibit barrier layer properties. The Nyquist plot (Figure 10) shows that the anodized samples in citric acid solutions have a lower corrosion rate than the non-anodized AA2024 aluminum alloy because they have depressed semicircles at high frequency (1 × 10^2^–1 × 10^4^ Hz).

**Figure 11:**
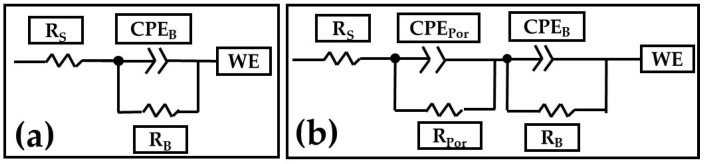
Electrical equivalent circuits from different samples. (**a**) un-anodized AA2024 and (**b**) citric–sulfuric acid-anodized samples with i = 3 and 4.5 A/dm^2^ immersed in 3.5 NaCl solution.

Figure 11a,b shows the equivalent electrical circuits (EEC) proposed to model the combination of kinetic processes from the EIS tests [67]. In this EEC, R_s_ is the solution resistance, R_Por_ is the porous layer resistance, and R_B_ is the barrier layer resistance. Furthermore, CPE_Por_ is the constant phase element relative to the porous layer, CPE_B_ is the barrier layer constant phase element, and WE is the working electrode. *n*_Por_ and *n*_B_ are the impedance exponents to the porous and barrier layers, respectively. The CPE can represent the roughness and heterogeneity of the porous and passive layers. The *n* coefficient can have several physical meanings at electrochemical interfaces, including surface roughness, corrosion products in the porous layer, and surface heterogeneity [68,69,70]. 

Table 3 presents the results obtained from the simulations using equivalent electrical circuits in Figure 11a,b. The variation in R_Sol_ is caused by coating morphology, which is linked to the surface charge and the electrochemical double layer [71]. The fit data of the EEC model that can be seen in Figure 9 and Figure 10 coincide largely with the experimental data. Therefore, in Table 3, the error presented by the simulations with EEC is less than <2.84 in most of the samples.

Additionally, the low values given by χ^2^ corroborate the accuracy of the proposed EEC model. They consider the porous layer resistance (R_Por_) of all the anodized samples with current densities 3 and 4.5 A/dm^2^. It can be established that this resistance also contributes to the corrosion resistance of anodized materials since samples 3A C1M S5, 3A C1M S10, 3A S1M, 4.5A C1M S10, and 4.5A S1M, R_Por_ are greater than the resistance of the barrier layer resistance (R_B_) presented by the non-anodized material. For samples 3A C1M, 4.5A C1M S5, and 4.5A C1M, R_Por_ was lower than the non-anodized material. These results show that the anodized material in a citric–sulfur acid solution protects against corrosion.

The higher values of CPE_B_ for AA2024 un-anodized, 3A C1M, and 4.5A C1M are attributed to a thinner barrier layer: the values of CPE_B_ are 1.27 × 10^−4^ F/cm^2^, 4.02 × 10^−5^ F/cm^2^, and 4.81 × 10^−4^ F/cm^2^, respectively. The results indicate that the thicknesses of these layers are more significant than the barrier layer formed naturally by the material un-anodized, AA2024 (see Table 3). According to some authors, the formed layer’s capacitance decreases as the formed coating’s thickness increases [72].

R_B_ is related to charge transfer resistance (R_ct_), which is inversely proportional to the corrosion rate in anodized materials. Analyzing the values of R_B_ of the samples 3A C1M, un-anodizing AA2024, and 4.5A C1M presented the lowest corrosion resistance in the evaluation medium with values of 6381 Ω·cm^2^, 13,920 Ω·cm^2^, and 19,478 Ω·cm^2^, respectively. In the samples 3A C1M S5, 3A C1M S10, 4.5A C1M S5, and 4.5A C1M S10, the R_B_ values were 5.81 × 10^6^ Ω·cm^2^, 0.36 × 10^6^ Ω·cm^2^, 0.14 × 10^6^ Ω·cm^2^, and 4.11 × 10^6^ Ω·cm^2^, respectively. The results indicate that the anodized citric–sulfuric acid mixtures have better corrosion resistance in the 3.5 wt. % NaCl solution than the material without anodized AA2024. For the samples 3A S1M and 4.5A S1M, R_B_ presented values of 6.20 × 10^6^ Ω·cm^2^ and 10.2 × 10^6^ Ω·cm^2^, respectively, providing excellent corrosion protection. It should be noted that this sample was created in a standard H_2_SO_4_ solution.

## 4. Discussion

All the anodized samples in the sulfuric and citric acid baths had superficial porosity, cracks, and practically the same morphology was presented for each solution regardless of the current density. SEM micrographs in Figure 1 and Figure 2 of the anodized AA2024 in citric–sulfuric acid show some cavities because of the copper intermetallic phase’s dissolution during anodizing in the different baths.

Cracks appeared on the anodic film formed on AA2024 due to extensive oxygen generation inside the film. The Al-Cu alloy contains the θ phase (Al_2_Cu), and the behavior of different second phases and the effect of the anodizing parameters on the properties of anodic films are critical for performing an effective anodizing process (see Figure 3 and Figure 4) [73]. Because of the high copper content, the AA2024 Al alloy had the lowest oxide thickness and number of cracks, which reduces the current efficiency (see Figure 6). The lower current efficiency is attributed to copper enrichment beneath the anodic film, copper oxidation, incorporation into the anodic film, and oxygen generation [74,75,76,77,78,79]. Aluminum combines with oxygen to form aluminum oxide during the anodizing process, which causes the oxide’s volume to grow and creates internal stress at the aluminum/oxide interface. When the oxide thickness and anodizing current density increase, so does this boundary stress. Additionally, when porosity decreases and the oxide barrier rises, higher interface stresses raise the anodic film’s hardness. However, when the material fails at the critical boundary stress, the stress releases and the fissures in the oxide film’s surface may be plainly seen. Additionally, the anodic voltage rises with current density, which results in a reduction in hardness because of the significant heat production. Finally, this compromised film structure causes the oxide’s hardness to decrease [80].

The coating is created during the anodizing process in the metal surface, and the current passes through the growing layer to reach the clean metal surface according to the coating thickness. The properties of the acid additives determine the electrolytes’ aggressiveness [79]. The literature has reported that working with low concentrations of sulfuric acid generates a dissolution of the anodizing layer [81]. The electrolyte is selected to carry out an anodizing process that significantly impacts the resulting anodic alumina film. Some film properties are determined by the type of electrolyte, its concentration, and its temperature [82,83,84]. One of the key factors affecting the oxide’s shape and chemistry is the electrolyte’s composition. One of the most prevalent additive mechanisms is the creation of complexes between organic molecules and aluminum. In this instance, the interaction of hard-ion carboxylates with trivalent aluminum cations results in easily formed complexes that are then absorbed into the anodic coating as insoluble metal soaps. The molecules are anticipated to protonate and become neutral in the solution without the inclination to migrate toward the anode since the pKa of carboxylic acids is higher than the pH value of the sulfuric acid baths [16,85]. The complexion additives form a thin film on the oxide surface protecting the metal [86].

Intermetallic particles strongly influence the morphology of the anodic layers in addition to their influence on oxide growth. According to their composition, copper-rich phases in the AA2024 Al alloy act as anodic or cathodic zones. S phases (Al_2_CuMg) serve as anodic zones, while θ phases (Al_2_Cu) serve as cathodic particles, causing pitting corrosion. A similar finding was made for the anodic layer formed on the 2214 Al alloy [87].

Copper intermetallic generates imperfections and fissures, and Vickers microhardness presents low values (see Figure 7). It has been demonstrated that Cu, Si, and Ni reduce the film’s hardness. [88]. When anodic oxides form in high-purity aluminum alloys, they are generally very dense and homogeneous, and the alloying elements can alter the properties of the layer formed, reducing corrosion resistance. The anodic oxides formed in aluminum alloys are not entirely composed of alumina due to the incorporation of alloying elements present in the composition of the substrate in the anodic layer and the incorporation of anions from the electrolyte. The effect of the alloying elements on the behavior and morphology of the anodized material depends on its nature. Based on this, the alloying elements can be classified into three categories. The first category is alloying elements with a lower Gibbs’ free energy of oxide formation compared to aluminum oxide (e.g., Mg, Li). The second group is made up of alloying elements that are more noble than aluminum and that oxidize during the anodizing process (e.g., Zn, Cu). Finally, the third group is made up of elements nobler than aluminum that do not undergo an oxidation process (e.g., Au) [89,90]. Since the most widely used alloys in the aerospace industry contain Mg, Cu, and Zn, attention must be paid to the effect of these elements on the oxide layer formed during anodizing.

Mg, being less noble than aluminum, oxidizes rapidly during the anodizing process, preferably forming MgO, since the volume occupied by MgO is less than the original volume occupied by elemental magnesium; the resulting oxide film is not continuous and tends to detach [91,92]. When Cu and Cu intermetallic are present on the aluminum substrate, the copper species are incorporated into the barrier-type anodic film as CuO units. In the first step, aluminum is preferentially oxidized at the oxide/alloy interface. The copper present, both in solid solution and in intermetallic phases such as the S phase (Al_2_CuMg) and θ phase (Al_2_Cu), does not oxidize. Therefore, it accumulates under the anodic film, leading to an area of copper enrichment. When a certain threshold of copper content is reached, the copper is oxidized and Al_2_O_3_, MgO, and CuO are simultaneously formed. Aluminum oxide, on the other hand, occupies a larger volume compared to elemental aluminum and, therefore, can fill the voids of the magnesium oxide, creating a continuous and well-adhered oxide film [93].

Cyclic potentiodynamic polarization curves (CPP) are used to describe and characterize anodic layers. After severe anodizing, Al alloys’ corrosion current densities fall considerably, and their polarization resistances rise (Figure 8 and Table 2). The intermetallic phases’ various types of flaws and the thickness of the oxide layer impact the anodic layer’s corrosion capabilities.

The corrosion potential of the anodized AA2024 alloy is more negative than samples anodized with citric–sulfuric acid, indicating enhanced anodic activity. The low corrosion resistance of anodized samples 3A C1M and 4.5A C1M is due to fewer cracks and oxide layer defects. This coincides with previous results where the oxide layer formed in an aluminum alloy 2618 was evaluated [94].

Anodized samples with current densities of 3 and 4.5 A/dm^2^ in various citric–sulfuric acid solutions exhibited acceptable electrochemical performance, which may be attributed to hard-ion carboxylate interactions with the Al^3+^ contained in the anodizing solution. This reaction results in intricate products being integrated into the surface, preventing corrosion [88]. The capacity of organic acids to adhere to metal surfaces and create a protective coating gives them their protective function [95].

The H_2_Cit^−^, HCit^2−^, and Cit^3−^ species are surface complexing ligands in the citric acid solution. The pH affects how much of these ligands is present in the solution (for example, HCit^2−^ species predominated in citric acid solution pH 6). When citric acid solutions come into contact with an aluminum oxide surface, multiple surface coordination events occur, resulting in various aluminum–citrate and/or aluminum–hydroxo–citrate surface species. The nature and structure of these surface complexes are primarily unknown. However, several writers have documented the presence of two six-membered chelate ring structures on the surface [96,97]. Citrate anions are adsorbed on the oxide surface and integrated into the structure of the oxide layer as Al-citrate complexes during the anodic oxidation of aluminum in citric acid and/or citrate solutions. Equations (1) and (2) show the possible surface coordination reactions that can occur on the surface of aluminum oxide in citric acid solutions with a pH range of 3 to 6 [98,99].

(1) $$\mathrm{Al}\;-{\mathrm{OH}}_{2}^{+}{+\mathrm{HCit}}^{2-}\;↔\;{\mathrm{AlHCit}}^{-}+\;{\;H}_{2}O$$

(2) $$\mathrm{Al}\;-{\mathrm{OH}+\mathrm{HCit}}^{2-}\;↔\;{\mathrm{AlHCit}}^{-}+\;{\mathrm{OH}}^{-}$$

There are two distinct steps in the pore nucleation process of the anodization in citric acid: First, a rapid flat barrier-type alumina film is created by simultaneously depositing alumina at the oxide–electrolyte contact and forming it at the metal–oxide interface. The anodizing process current density creates an electric field, which causes O^2−^/OH^−^ ingress from oxide–electrolyte to metal–oxide surfaces and Al^3+^ egress from metal–oxide to oxide–electrolyte interfaces. Al–citrate complexes, which stabilize a specific quantity of Al^3+^, are present in the electrolyte.

Second, the electrolyte’s Al–citric complex slowly converts to citric acid-incorporated alumina and deposits it unevenly on barrier-type alumina, which causes an electric field concentration between protuberances and pore development. It should be noted that the protuberances created on the formed alumina surface because of the citric acid’s random and gradual incorporation of alumina are what cause the electric field to concentrate in certain areas. Alumina is quickly generated at the oxide–metal interface because of field-assisted dissolution occurring at the oxide–electrolyte surface, which thins the flat barrier layer that has been formed. To maintain the equifield field strength and cause the pore-forming, oxide growth at the metal–oxide interface with a scallop shape is controlled by the morphology at the oxide–electrolyte interface [52].

The EIS results indicate that the highest resistances found in citric–sulfuric acid solutions were in samples 3A C1M S5 and 4.5A C1M S10, with resistances of 5.82 × 10^7^ Ω·cm^2^ and 4.11 × 10^7^ Ω·cm^2^, respectively, which are excellent when compared with works published by other authors. Regarding the EIS results, resistances from 2 × 10^5^ Ω·cm^2^ to 10.7 × 10^6^ Ω·cm^2^ have been found in various previous works to show the hard anodizing of 6061 aluminum using a pulsed current [100]. In comparison, barrier layer resistances from hard anodizing aluminum alloy 7075 range from 6.18 × 10^7^ Ω·cm^2^ to 1.11 × 10^9^ Ω·cm^2^ [93]. In some additional works of hard anodizing with a pulse for anodized aluminum alloys of high purity, resistances of 8.53 × 10^9^ Ω·cm^2^ were discovered. In contrast, anodized aluminum alloy 2024 showed resistances in citric–sulfuric acid combinations between 1.38 and 2.44 × 10^5^ Ω·cm^2^ [101]. The morphology of porous anodic films on AA2024 alloy is significantly influenced by copper enrichment, the presence of copper and oxygen species in the film, the release of oxygen gas into the electrolyte, or a combination of these factors [48]. These results show that citric acid-based solutions can be used to hard anodize AA2024 aluminum alloys, resulting in resistance values that are on par with or higher than those published in the literature.

Numerous investigations have demonstrated that the barrier layer qualities are reflected in the low-frequency range, whereas the porous layer properties are reflected in the high and medium frequency ranges [102,103,104,105]. According to some writers, the anodic coating on aluminum comprises two layers: a very thin, compact barrier layer and a larger, porous layer, the latter of which comprises pores and walls made of hexagonal-shaped cells. The porous anodic film can be simulated using a variety of equivalent circuits, and these models have been effectively used to explain the characteristics of numerous materials’ porous layers and compact barriers [67,102,106,107,108,109,110,111].

## 5. Conclusions

- Porous anodic alumina films were successfully produced under hard anodizing conditions on AA2024 alloy. Intermetallic phases in aluminum alloys influence the anodic layer growth rate and morphology. Oxide layers formed on AA2024 alloys with coarse intermetallic phases contained large cavities and surface defects.
- Anodic films with porosity, cracks, and lateral porosity, commonly encountered during anodizing of AA2024 alloy, were obtained. In addition, oxidation of the second phase particles is achieved, and, as a result, its consumption generates cavities on the surface of the films and in the cross-section.
- The thickness and Vickers microhardness obtained in the hard anodic coatings were low due to secondary phases rich in copper that prevent the film’s homogeneous growth. These secondary phases are associated with the generation of lateral porosity that also decreases properties. Lower thickness and microhardness are presented because of the formation of large cavities and defects induced by the activity of the Cu and Fe coarse intermetallic phases.
- The cyclic potentiodynamic polarization technique indicated that higher E_corr_, and lower corrosion current densities (i_corr_), were presented in samples 3A CIM S5 and 4.5A C1M, representing that this sample provides more corrosion resistance than conventional sulfuric acid anodizing or the un-anodized alloy.
- The EIS results indicate that hard-anodized coatings with citric–sulfuric acid showed resistance when exposed to a 3.5 wt. % NaCl solution for the samples 3A CIM S5 and 4.5A C1M S10.
- Type III hard anodizing is possible with mixtures of citric–sulfuric acid solutions, which will present good mechanical properties and greater corrosion resistance than the material without anodizing.

## Figures and Tables

**Table 1:** Anodizing process parameters and sample nomenclature.

Material	Anodizing				Sealing Process	Samples
	Current Density (A/dm^2^)	Time (min)	Bath for Anodizing			
			Sulfuric Acid	Citric Acid		
AA2024	3	60	5 mL/L	1 M	Deionized water Temperature at 95 °C Time 60 min	3A C1M S5
			10 mL/L	1 M		3A C1M S10
			-	1 M		3A C1M
			1 M	-		3A S1M
	4.5	60	5 mL/L	1 M	Deionized water Temperature at 95 °C Time 60 min	4.5A C1M S5
			10 mL/L	1 M		4.5A C1M S10
			-	1 M		4.5A C1M
			1 M	-		4.5A S1M

**Table 2:** Electrochemical parameters using CPP of AA2024 and different anodized samples and immersed in NaCl solution.

Sample	E_corr_ (V)	E_pit_ (V)	E_A–C_ (V)	I_pass_ (A/cm^2^)	i_corr_ (A/cm^2^)	Hysteresis
AA2024	−0.656	−0.656	−0.895	-	3.43 × 10^−7^	Positive
3A C1M S5	−0.600	−0.430	−0.791	-	1.57 × 10^−7^	Positive
3A C1M S10	−0.598	−0.308	-	1.38 × 10^−8^	1.14 × 10^−8^	Negative
3A C1M	−0.563	−0.242	−0.903	-	2.31 × 10^−5^	Positive
3A S1M	−0.293	−0.293	0.298	6.77 × 10^−9^	1.36 × 10^−10^	Negative
4.5A C1M S5	−0.446	0.334	-	7.52 × 10^−6^	2.35 × 10^−8^	Positive
4.5A C1M S10	−0.488	0.034	−0.774	2.44 × 10^−6^	1.26 × 10^−9^	Positive
4.5A C1M	−0.593	−0.593	−0.917	-	2.55 × 10^−6^	Positive
4.5A S1M	−0.180	−0.180	-	1.87 × 10^−8^	8.30 × 10^−11^	Negative

**Table 3:** Electrochemical parameters using EIS data obtained from the un-anodized AA2024 and anodized samples immersed in NaCl solution.

Samples	R_Sol_ (Ω·cm^2^)	CPE_Por_ (F/cm^2^)	R_Por_ (Ω·cm^2^)	*n*_Por_	CPE_B_ (F/cm^2^)	R_B_ (Ω·cm^2^)	*n*_B_	Error	χ^2^
AA2024	28.5	-	-	-	1.27 × 10^−4^	13,920	0.80	˂1.42	1 × 10^−2^
3A C1M S5	28.78	2.28 × 10^−7^	46,014	0.78	9.30 × 10^−7^	5.81 × 10^6^	0.81	˂1.96	1 × 10^−2^
3A C1M S10	14.26	8.80 × 10^−7^	47,545	0.74	3.12 × 10^−6^	0.36 × 10^6^	0.69	˂1.14	1 × 10^−2^
3A C1M	22.77	1.41 × 10^−6^	560	0.81	4.02 × 10^−5^	6381	0.64	˂1.95	3 × 10^−3^
3A S1M	24.40	6.68 × 10^−7^	152,820	0.77	8.41 × 10^−7^	6.20 × 10^6^	0.94	˂1.79	1 × 10^−2^
4.5A C1M S5	63.58	1.13 × 10^−7^	2677	0.85	6.11 × 10^−6^	0.14 × 10^6^	0.54	˂2.84	1 × 10^−2^
4.5A C1M S10	16.88	2.27 × 10^−6^	15,651	0.75	1.26 × 10^−6^	4.11 × 10^6^	0.90	˂2.04	3 × 10^−2^
4.5A C1M	25.58	5.43 × 10^−5^	6555	0.74	4.81 × 10^−4^	19,478	0.71	˂2.25	1 × 10^−3^
4.5A S1M	25.35	6.55 × 10^−7^	89,049	0.61	1.26 × 10^−6^	10.2 × 10^6^	0.91	˂2.17	7 × 10^−3^

## Data Availability

Not applicable.
